# An IoT-Focused Intrusion Detection System Approach Based on Preprocessing Characterization for Cybersecurity Datasets

**DOI:** 10.3390/s21020656

**Published:** 2021-01-19

**Authors:** Xavier Larriva-Novo, Víctor A. Villagrá, Mario Vega-Barbas, Diego Rivera, Mario Sanz Rodrigo

**Affiliations:** ETSI Telecomunicación, Universidad Politécnica de Madrid (UPM), Avda, Complutense 30, 28040 Madrid, Spain; victor.villagra@upm.es (V.A.V.); mario.vega@upm.es (M.V.-B.); diego.rivera@upm.es (D.R.); mario.sanz@upm.es (M.S.R.)

**Keywords:** Internet of Things, machine learning, intrusion detection system, preprocessing techniques, traffic categorization

## Abstract

Security in IoT networks is currently mandatory, due to the high amount of data that has to be handled. These systems are vulnerable to several cybersecurity attacks, which are increasing in number and sophistication. Due to this reason, new intrusion detection techniques have to be developed, being as accurate as possible for these scenarios. Intrusion detection systems based on machine learning algorithms have already shown a high performance in terms of accuracy. This research proposes the study and evaluation of several preprocessing techniques based on traffic categorization for a machine learning neural network algorithm. This research uses for its evaluation two benchmark datasets, namely UGR16 and the UNSW-NB15, and one of the most used datasets, KDD99. The preprocessing techniques were evaluated in accordance with scalar and normalization functions. All of these preprocessing models were applied through different sets of characteristics based on a categorization composed by four groups of features: basic connection features, content characteristics, statistical characteristics and finally, a group which is composed by traffic-based features and connection direction-based traffic characteristics. The objective of this research is to evaluate this categorization by using various data preprocessing techniques to obtain the most accurate model. Our proposal shows that, by applying the categorization of network traffic and several preprocessing techniques, the accuracy can be enhanced by up to 45%. The preprocessing of a specific group of characteristics allows for greater accuracy, allowing the machine learning algorithm to correctly classify these parameters related to possible attacks.

## 1. Introduction

Cyberspace plays a fundamental role in the society and economy, as the Internet has changed the means of communication for people or organizations. Furthermore, different devices, applications, and services which are linked to cyberspace are included inside the term Internet of Things (IoT). So, information communication technology (ICT) has been intensively applied for the deployment of various types of sensors actuators, for machine-to-machine (M2M) communications infrastructures [1], with the principal objective of processing the huge amount of data that is provided for these services or applications.

According to the process of gathering information, transmission, and processing from IoT systems, IoT has an entity-based architecture that is divided in three main layers. These layers are usually named: terminal perception layer, network transport layer, and application service layer [2]. The terminal perception layer is composed by the source of IoT data collection. The units involved in this layer include physical entities representing real devices units (sensors devices, identification devices, tracking and positioning devices). Furthermore, the network transport layer transmits the information gathered by the perception layer to the application service layer. Finally, the application service layer process the data transmitted from the network transport layer to various industries or entities, providing services for different users through different fields, such as smart grids, smart homes, and smart cities [2].

**Figure 1:**
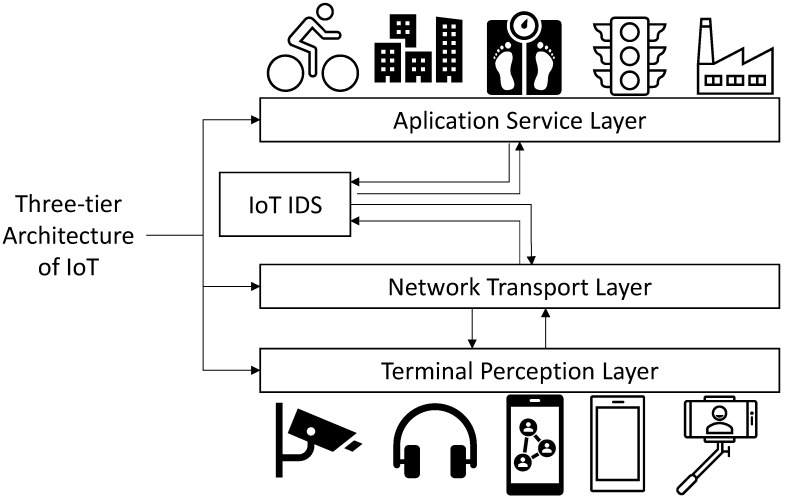
Three-tier architecture of IoT and IoT IDS approach.

The network transport layer can suffer several security threats, such as distributed denial of service (DDoS) attacks, sending traffic and consuming network and computing resources [3]. Additionally, the application layer from IoT systems is vulnerable to several types of cybersecurity attacks, such as Worms, Trojan, DoS, or Spyware. These types of attacks are becoming more sophisticated, increasing its number day to day [4]. Additionally the application of different standardized communications combined with the limited computer power and the high number of connected devices, can make the traditional security countermeasures not efficient in IoT systems [5]. This research is focused on the detection of possible attacks between the transport network layer and the application layer as presented in Figure 1, which is detailed through this research.

For this reason, developing security solutions for IoT is indispensable, with the objective to prevent and mitigate possible cybersecurity issues.

To detect IoT attacks on the network transport layer, networks intrusion detection systems (NIDS) have been deployed as a second line of defense after Firewalls, antivirus, and access control systems [6] for connected smart things. A NIDS is a software with functionalities focused on monitoring ICTs from fraudulent uses, unauthorized access or any other cyberattack. There exist different types of NIDS, including those based on signature detection and those aimed at detecting anomalies. NIDS oriented to anomaly detection are able to perform an examination of the network, verifying its behavior and activity, then being able to detect and catalog possible deviations of patterns that represent the malicious behavior of possible cyberattacks. Although such systems represent a robust solution for attack detection, they must deal with a major challenge that deteriorate their accuracy: the detection of false positives due to the similarities between legitimate and anomalous observations.

Thus, Machine Learning techniques, specifically deep learning (DL) algorithms, are being proposed as an effective solution for dealing with this problem [7,8]. One of the most important requirements of NIDS based on DL techniques is the preprocessing phase, which can affect the accuracy of an algorithm in a significant way [9]. This data preprocessing consists of transforming the input data by using different techniques such as One-Hot encoding [10], z-score [11], and standardization type min-max [12]. However, IoT ecosystems are delocalized, distributed and composed by a large number of devices with computing performance limited in resources. IoT is also limited by network bandwidth capacity. This implies that NIDS based on anomalies must be efficient and accurate. In this way, this article aims to identify the preprocessing level configuration that yields the best accuracy of the underlying model.

In addition, and taking into account that IoT systems try to reduce the computational cost as much as possible, strengthening the learning model and avoiding the possible overfitting to increase the efficiency of the underlying learning models, this research work proposes the use of the categorization defined in [13]. This categorization is composed by four groups of features which include basic connection features, content characteristics, statistical characteristics, and finally, a group which is composed by traffic-based features and connection direction-based traffic characteristics. The objective of this research is to evaluate this categorization by using various data preprocessing techniques based on transforming categorical values into numerical values and by applying standardization and normalization.

Finally, up-to-date benchmark datasets for IoT IDSs are currently almost non-existent [6,8], although some datasets have been generated for this environments, e.g., the IoTID20 [14]. As a consequence, for this research we have opted to use three of the most widely accepted and adopted benchmark datasets, such as KDD99 [15] and NSL-KDD [16], which is an improved version of the KDD99. Additionally, we use UNSW-NB15 [17], and UGR16 [18], a novel dataset which has been applied previously in the study of NIDS based on anomalies [9].

This paper introduces the background and related work to this research work during Section 2. Additionally, the paper introduces the proposal of a new way of data preprocessing based on traffic characterization for IDSs in Section 2. Furthermore, in Section 3, Section 4, Section 5 and Section 6, we explain the problem statement and the proposed methodology, defining the methodology ML and preprocessing model presented in this paper. These sections also include the architecture proposed for applying the multilayer perceptron (MLP) and the obtained results after applying our proposal to the UNSW-NB15, UGR16, and KDD99 datasets. Finally, Section 5 includes the discussion of the main conclusions and future lines for this research.

## 2. Background and Related Work

### 2.1. Data Threatment

Data preprocessing applied to NIDS based on ML algorithms is divided into three main categories: data reduction, treatment of missing data, and data scaling.

Data reduction can be divided into feature selection and case selection. Feature selection is the process of selecting a subset of features that provide a similar impact in the results, rather than selecting the entire set of features of the selected dataset. Its main goal is to increase the accuracy of a ML algorithm and reduce the cost for the fitting and validation in terms of computer resources [19]. Case selection is similar to feature selection, being their main difference that case selection intends to identify and remove the redundant data from the dataset. This method allows to reduce the size of the dataset from its original dimensions, reducing the time required for the algorithm to be fitted and validated [20].

Treatment of missing data is also divided in data elimination and data imputation. The first one is composed by removal by lists and by pairs, while data imputation is divided into: mean imputation, hot-deck imputation, cold imputation and regression [21].

Data scaling is defined by the transformation of the data using diverse methods, i.e., standardization and normalization. This type of processing allows to transform the data from an established scaling function. Consequently, the values of a class of a dataset expresses the same degree of influence for the ML algorithm [22]. Taking this idea, some research works have introduced other aspects of preprocessing techniques based on four main components: feature selection, feature reduction, clustering, and hybrid approaches [23]. Nevertheless, these preprocessing techniques were only approached through the use of the KDD99 dataset.

### 2.2. Preprocessing Related Works and Network Iintrusion Detection Systems Data Preprocessing

Several studies have been carried out in the area of data preprocessing, with the aim of optimizing the data used by different ML algorithms and ultimately to enhance the accuracy with less computing performance.

In this sense, Ref. [24] introduced different anomaly detection algorithms which were applied to the NSL-KDD dataset to evaluate different preprocessing techniques. The research applied standardization and normalization to the dataset obtaining an overall accuracy of 99%. The research work presented in [25] evaluated the impact of different attribute normalization schemes on a combination of different features from the dataset NSL-KDD. The authors provided an evaluation of three different algorithms using six different characteristics where a K-nearest neighborhood obtained the best accuracy with 98.9%, followed by a multilayer perceptron model with 96.5% and naïve Bayes with 93.3%. The study remarked the importance of the attribute selection and normalization to increase the accuracy of the models. Authors in [26] proposed an NIDS based on anomaly detection, where categorical values were mapped into numerical values, while the non-categorical values were normalized in ranges between zero and one. Applying this procedure, the authors highlighted an improvement in the accuracy of the model proposed, achieving a maximum accuracy of 99.5% in denial-of-service attacks. The study conducted in [27] presented an evaluation of various configurations of data preprocessing. The authors proposed a standardization of type z-score of 12 attributes and 34 attributes normalization obtaining a maximum accuracy of 98% for the standardization model and 99% for the normalization model. The research proposed the use of a back propagation neural network: the concluding results introduce the standardization and selection of hyperparameters as the principal characteristics for enhancing the accuracy. Additionally, the mentioned research used the dataset KDD99.

As observed, different studies on the optimization of preprocessing data have been carried out, traditionally, by using the KDD99 or NSL-KDD dataset. In [28], authors presented a study that determined that the most commonly used datasets for the different analysis of NIDS based on anomalies detected by ML techniques are the KDD99 with 63.8% of popularity followed by the NSL-KDD with 11.6%. In addition, this study proposed the application of an ML-based NIDS model for the detection of anomalies in IoT systems. In this sense, this research introduces the analysis of characteristics and preprocessing models based on normalization and scaling for two new benchmark datasets, the USW-NB15 and the UGR16. The research carried out and presented in this paper tries to identify the most suitable characteristics for developing ML-based NIDS models, reducing the processing time, and improving the accuracy for IoT environments.

## 3. The Proposed Approach

**Figure 2:**
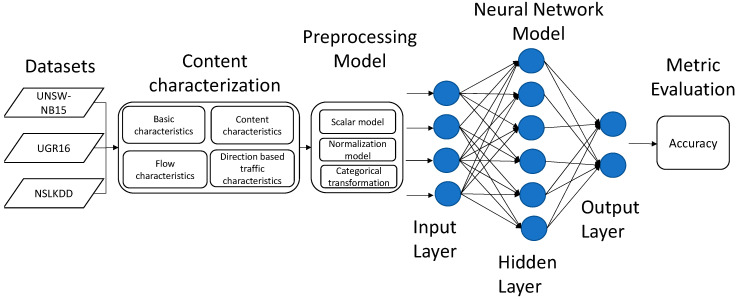
Categorical data preprocessing results in terms of accuracy scaled between 0 to 1.

The proposed work presented in this research is designed to evaluate the set of characteristics proposed in [13]. The architecture proposed for the IDS consists in the introduction of individual preprocessing techniques based on a content characterization. The system is presented in Figure 2. As can be seen in the figure, it is composed by some phases for the evaluation in order to obtain the best accuracy. These stages are described in the following sections.

### 3.1. Datasets under Study

In relation to the datasets created for IoT networks, many studies use the KDD’99 dataset. This dataset has become a reference for various investigations related to NIDS for IoT networks [5]. However, in cybersecurity, this dataset is currently considered obsolete, due to the age of attacks it presents [9,13]. Several studies have compared different cybersecurity datasets, each one of these datasets being created through different methods, in the field of IDS. Also, nowadays, these datasets have been considered as benchmark datasets for the evaluation of IDS, and also applied to IoT IDS [4,9,16,17,18,24,29,30]. Therefore, an updated dataset with current attacks, UGR16 and real collected traffic with up-to-date attacks, is proposed as the basis for the analysis in this research. Additionally, to extend the analysis comparison, it is proposed to use two benchmark datasets, i.e., UNSW-NB15 and NSL-KDD.

#### 3.1.1. Dataset UGR16

The UGR16 [18] dataset is a more realistic attempt made at capturing NetFlow traces covering more than four months of network traffic from an internet service provider (ISP). An important advantage of this dataset is the normal traffic, that was adequately captured from different sensors located in the ISP networks. This dataset takes around 19.900 million of unidirectional flows offering a big scope for experimentation. Additionally this dataset is clean from synthetically generated attacks [18,31]. The UGR16 has 13 characteristics: timestamp of the end of a flow (time), flow duration (duration), source IP address (sip), destination IP (dip), source port (source port), destination port (destination port), protocol (protocol), flags (flags), forwarding status (forward_status), type of service (type_service), packets exchanged in the stream (pack_exchanged), their corresponding number of bytes (bytes), and the attack type (attack_tag).

There are multiple options for obtaining portions of the UGR’16 dataset. In this case, the week of 2 August, test version, was taken as a sample. This was selected for the present study, due to the fact that the test versions have synthetic traffic which allows applying more varied data. The original version of the data set is 81 GB. For this research, a 1.4 GB portion was selected, making it proportional to the original dataset in terms of attack and normal traffic proportions. Table 1 shows the relationship between normal traffic and attacks from the UGR16 dataset.

All the features mentioned above were considered for the proposed model, except for the time feature. That feature was eliminated since the evaluation of the NIDS as a model based on time series is not within the scope of this research. Therefore, the research was carried out with 12 basic characteristics.

#### 3.1.2. Dataset UNSW-NB15

The UNSW-NB15 [17] dataset has 49 features classified into 5 categories: flow characteristics, basic characteristics, content characteristics, time characteristics, and generated additional characteristics. Table 2 shows the relationship between normal traffic and attacks from the UNSW-NB15 dataset

- The basic characteristics, unlike in NSL-KDD, include the attributes that represent the protocol connections, most of them similar to the basic characteristics in NSL-KDD.
- The flow characteristics include the identifying attributes between the hosts.
- Content characteristics involve TCP/IP attributes and some http connections.
- The time characteristics contain all the attributes related to time i.e., arrival time between packets.
- Additional characteristics generated divided into two groups, i.e., general purpose to protect the service of protocols and connection characteristics.

#### 3.1.3. Dataset NSL-KDD

The NSL-KDD dataset is an improved version of the KDD99. It not only solves the redundant records problems of the KDD99, but also makes the number of traces appropriate in the training and testing dataset [32]. This clean version prevents the machine learning algorithm from being biased during the training data phase [33]. Finally, thanks to this dataset and the high number of studies from the last decade, we can still make a comparison for suitable algorithms related to the last decade. The NSL-KDD dataset has 42 characteristics, classified into 3 categories: basic characteristics, content characteristics, and traffic characteristics. Table 3 shows the relationship between normal traffic and attacks from the NSL-KDD dataset.

- The basic characteristics include all the attributes that can be extracted from an individual TCP/IP connection.
- The content characteristics consist of some specific characteristics necessary to detect attacks that show suspicious behavior in the data portion, for example, number of failed login attempts.
- Flow characteristics include the characteristics calculated with respect to a window interval.

### 3.2. Data Preprocessing

Data preprocessing consists in transforming the data values of a certain dataset, aiming to optimize the information acquisition and process. Normally, there is a very large contrast between the maximum and minimum values of the dataset, so normalizing the data minimizes the complexity of the algorithm for its corresponding processing. According to [27], the normalization of the data allows an adequate benefit for the classification of algorithms related to neural networks. In this case, if the back-propagation technique is used in neural networks, the normalization of the input values will speed up the training phase, turning it into a more efficient neural network.

#### 3.2.1. Normalization Function

The main normalization function is based on data scaling, which consists of the min-max algorithm, which is capable of converting the current range of data typically in the interval [–1, 1] and [0, 1]. The normalization formula is presented in Equation (1).

(1) $$p=\frac{(\left(x-x_{min}\right)\left(max-min\right)}{(x_{max}-x_{min})+min}$$

where (*min*, *max*) is the specified range of input variable, (*x_min_*, *x_max_*) the initial range of values of input variables, and *p* is the converted input value.

#### 3.2.2. Standardization Function

The standardization function, or z-score, is able to normalize the features of the dataset. It has the properties to normalize the features values of a dataset, normalizing a standard distribution. These values are represented in Equation (2), where µ is the mean (the average value of a feature over all the values of that feature in the dataset) and ∂ is the standard deviation of the mean.

(2) $$x^{\prime \left(j\right)}=\frac{x^{\left(j\right)}-{µ}^{\left(j\right)}}{\partial^{\left(j\right)}}$$

### 3.3. Deep Learning Algorithm under Study

The multi-layer perceptron neural network (MLPNN) consists in a linear classification algorithm capable of ordering the input data into categories. MLPNN are feed-forward neural networks that consists of a large number of neurons classified into input units (input layer), output units (output layer) and hidden units (hidden layer). The weights assigned to the connections are estimated using a back-propagation algorithm. The values of the weights define the performance of the neural network.

In this article we only focus on supervised learning, due to the specific problem stated in it. The proposed model is limited to analyzing the sequence of the attacks because the objective of the study is not focused temporal time series. Thus, the main objective is to identify the optimization of characteristics proposed in [13] for diverse datasets at the level of preprocessing. For this, the MLPNN architecture proposed in this research was designed according to the criteria established in the aforementioned study, defining an MLP model and its associated best hyperparameters.

The configuration for the neural network taken as the basis for the comparison in this study consists of a 4-layer network. The input layer with a neuron density corresponding to the input data of each dataset, the hidden layers with a density equal to the rule that obtained the best precision in the research mentioned below, and an output layer with an equal neuronal density according to the attack classification are proposed in this research. The last layer was defined by a density equal to one, since a binary class classification between attack and no-attack will be obtained at the output. For the model proposed in this study, the initialization of the weights was done applying Glorot normal initializer [34] with no seed. Furthermore, the computational experiments were averaged with the function Earlystoping [35] setting the loss with a min_delta of 10^−3^ in order avoid the overtraining of the algorithm. The patience was set to 5 (number of epochs with no improvements after the training is stopped). This value was settled after several test in order to improve the computation costs of the training. Finally, the best weights were obtained setting the variable restore_best_weithgs to True which is capable to restore to the model from the epoch with the best value of the monitored loss

### 3.4. Evaluation Metrics

Accuracy (AC) is considered one of the most important performance indicators. As one of the most used metrics in several works as was presented during Section 2. This metric determines the number of records in a class predicted correctly. The value of true positive (TP) is equivalent to the correctly predicted values, corresponding to a class. The false positive (FP) value is the number of predictions that are not equivalent to the corresponding class. The true negative (TN) is the result of those values that are presented, corresponding to the number of records that are identified as normal. The false negative (FN) is the incorrectly predicted result for a corresponding class. Equation (3) shows how the AC and relationship of these parameters is calculated. Table 4 presents the corresponding confusion matrix.

(3) $$AC=\;\frac{TP+TN}{TP+TN+FP+FN}$$

## 4. Methodology and Experimentation

### 4.1. Entire Set of Characteristics Evaluation

Each dataset considered for performing the experimentation of this research is composed by different characteristics presented during Section 3. The main proposal at this point is to determine the best data preprocessing function by proposing the transformation of categorical variables into numerical data and the standardization and normalization functions for the underlying model.

The neural network algorithm proposed for this case study does not allow the use of text-type input variables, so these variables are transformed into binary vectors by the one-hot encoding [36] method and the attacks were transformed into binary vectors. Table 5 presents the proposal of non-numerical data that were transformed using the different methods mentioned above.

All the datasets were divided into training and testing datasets where the 75% of the entire dataset was considered for the training and the 25% for the testing. These datasets were applied with the function of train_test_split [37] with defined random state [38] variable for all the datasets.

As a first point of analysis, the study is carried out using the proposed datasets submitted to the same architecture of the neural network, without pre-processing the data. Only the categorical variables shown in Table 6 were transformed. This was carried out to obtain a base precision measure, which would allow us to determine the increase in precision based on the various types of preprocessing techniques applied.

**Figure 3:**
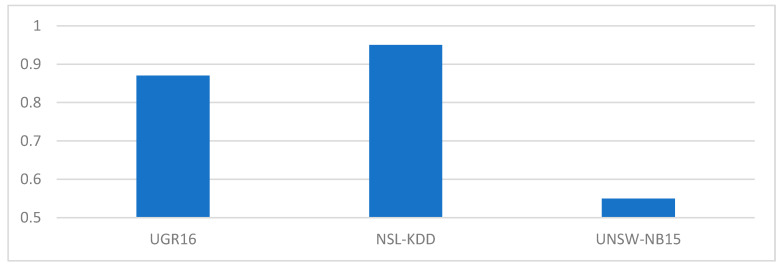
Categorical data preprocessing results in terms of accuracy scaled [0–1].

The results obtained from the training and validation of the proposed algorithm show that the NSL-KDD dataset offers a better accuracy, with 95.5%, compared to 87.68% of the UGR16 and 55.80% of the UNSW-NB15. These values are represented in Figure 3. It should be noted that the architecture of the neural network is the same for all datasets as described in Section 3.3.

In order to carry out the evaluation of the proposed preprocessing methods, the first approach consists in evaluating the total set of features for each individual dataset, testing them with the same preprocessing technique. The total set of features evaluated with the standardization function corresponds with the label z_score_all, following with the normalization functions: minmax_0_all for the configuration with a minimum of 0 and a maximum of 1 and minmax_1_all for the configuration with a minimum of −1 and a maximum of 1.

The evaluation presented an increase in terms of accuracy for each individual dataset. In case of UGR16, an accuracy of 99.3% was obtained by applying z_score_all; while the accuracy was increased to 99.88% when applying the minmax_0_all configuration; and correspondingly for the minmax_1_all configuration the accuracy was reduced to 99.18%.

The same methodology was applied for the NSL-KDD dataset with the configurations: z_score_all, minmax_0_all, and minmax_1_all, where values of 97.89%, 96.25%, and 96.48% of accuracy were obtained, respectively.

**Figure 4:**
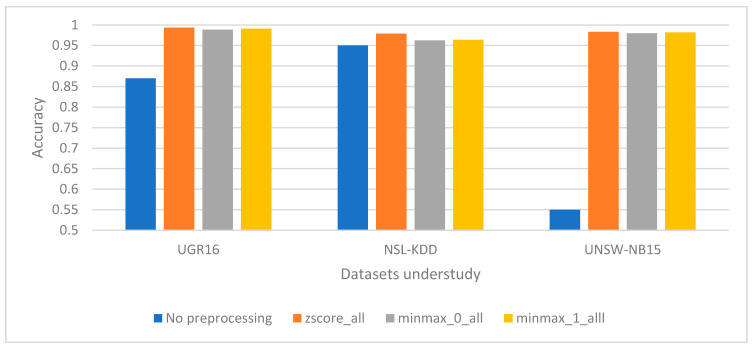
Standardization and normalization functions applied for the entire group of characteristics versus no processing functions for the UGR16, NSL-KDD and UNSW-NB15 datasets accuracy scaled between 0 to 1.

Finally, the UNSW-NB15 dataset was evaluated with the same configuration mentioned above, that is, z_score_all, minmax_0_all, and minmax_1_all, and producing values of 98.3%, 98%, and 98.2% in terms of accuracy respectively. All of these results are summarized in Figure 4.

The experimentation exposed that the preprocessing techniques could enhance up to 45% the accuracy in respect to the no preprocessing techniques, such as the case for the UNSW-NB15 dataset. Furthermore, it can be seen that z_score_all configuration technique for all groups of characteristics provides better results for all the datasets under study.

### 4.2. Individual Set of Characteristics Evaluation

As mentioned before, the objective of this research work proposes the use of the categorization defined in [13] which is defined by four groups characteristics. This categorization was applied to the entire set of features of each proposed dataset under study mentioned previously. Additionally, three preprocessing techniques were applied individually for each individual set of characteristics, in order to compare the results. Specifically, the standardization (z-score), normalization (min–max), and no preprocessing (-) technique were applied.

In the case of the UGR16 dataset, since it only has 12 basic characteristics, three evaluations were carried out, which were those mentioned in Section 4.1 and exposed in Figure 2. In the case of the NSL-KDD and UNSW-NB15 datasets, a complete comparison of preprocessing techniques was possible because they present diverse groups of characteristics, as it was shown in Section 3. Thus, results obtained for NSL-KDD and UNSW-NB15 are presented and analyzed.

Table 6 shows the 10 main accuracy variations with the most favorable results obtained, arranged in descending order applying the variations of the data preprocessing techniques for each of the groups of characteristics of the NSL-KDD dataset. These data were characterized according to the proposal in [13]. However, this dataset does not contain direction-based traffic characteristics, so the evaluation of preprocessing techniques was done without this group of characteristics.

Table 7 shows the accuracy variations of the applied preprocessing techniques for the various sets of characteristics presented in the UNSW-NB15 dataset, presenting the 10 best variations ordered in descending order according to the validation score.

Table 8 shows the best configurations obtained with the highest precision for each dataset (UNSW-NB15, NSL-KDD and UGR16) by groups of characteristics. This table shows that the standardization to the group of basic characteristics and statistical traffic characteristics allows increasing the accuracy of the algorithm. Unfortunately, a direct comparison between the content characteristics cannot be obtained, since for the UNSW-NB15 dataset and for the NSL-KDD dataset their preprocessing gives a non-significant precision variation. Since the UGR16 dataset contains only basic characteristics, it allows determining a substantial increase in precision with the use of the standardization algorithm.

Additionally, we have evaluated the most accurate models for each dataset i.e., N01, N05 and N07, presented in Table 8. These models were evaluated with the confusion matrix in term of percentage as is presented in Table 9. The percentage was considered related to the total number of flows from each dataset previously evaluated. Finally, these models were also evaluated with other metrics such as precision and recall as presented in Table 10.

## 5. Discussion

The preprocessing of the basic characteristics allows greater accuracy, because a mean distribution of the values is generated, allowing the ML algorithm to correctly identify these parameters related to attacks.

Research works presented in Section 2 were analyzed looking for the existence of preprocessing techniques such as scalar, normalization and categorical transformation. The comparison presented in Table 11 exposes the best results obtained in terms of accuracy between the works mentioned before and our proposed models for each one of the analyzed datasets in through this research.

The results obtained determine that our proposal gets better results with the preprocessing techniques commonly used and the assumed group of characteristics. Most of the recent studies have been developing models for benchmarks datasets such as KDD99 or NSL-KDD. In this way, the principal difference is that our proposal enhances the detection rate in terms of accuracy for individual groups of characteristics through different datasets. Furthermore, this characterization may improve a comparison in terms of precision between different benchmark and non-benchmark datasets taking into account the similarity of the proposed NetFlow characterization of characteristics.

Thus, the NIDS based on ML presented in this paper offer several advantages over other models. First, it is able to easily detect an attack, correctly differentiating it from normal traffic. Second, thanks to the use of benchmark and up-to-date datasets, the proposed model is capable of facing sophisticated attacks. These advantages, together with the classification proposed in [13] have allowed the proposed ML model to improve the accuracy, which is essential for its deployment in real environments.

After performing the experiments described above with the proposed neural network model, it is possible to obtain an acceptable precision greater than 99% in each of the datasets used. This shows the benefit of data preprocessing based on the categorization that we propose, demonstrating that a data standardization based on the scaling of variables using the z-score algorithm allows increasing the precision of the ML algorithm for the model.

Concretely, a high precision was obtained with the proposed model and the various types of preprocessing models. Table 7 shows the diverse configurations applied and the enhance up to 44% in the accuracy of the algorithm when preprocessing the information properly, this specifically in the case of the UNSW-NB15 dataset. The actual variation in precision depends on the ML model proposed and its hyperparameters, so this study focuses on the application of a single model to the various datasets, which is a way of remark the contribution of the preprocessing techniques proposed by group of characteristics.

## 6. Conclusions and Future Lines

This research work introduces new comparisons between the use of various datasets such as the case of UNSW-NB15 and UGR16, allowing to extend the various studies in the application of ML algorithms for the case of anomaly-based NIDS. In all the datasets previously studied, the transformation of categorical values to numerical values is essential, as this ensures that the data can be correctly coupled to the ML algorithm. Furthermore, the study shows that data preprocessing task is very important for the ML algorithm to obtain greater precision in terms of the classification of anomalies, as can be seen by comparing Figure 3 and Figure 4.

The most relevant conclusion that this study provides is the importance of preprocessing characteristics, such as basic characteristics and statistical traffic characteristics using z-score standardization techniques, which allows increasing precision since it allows using the mean deviation of the variables. As future actions, we will extend this research and we intend to apply the proposed model and preprocessing functions by individual group of characteristics into a real environment, with real data collected from IoT systems, such as the platform of Smart City services proposed in [1], to demonstrate the efficiency of our implementation. This implementation could be done with technologies such as Structured Streaming from Spark [39], which is able to replicate the neural network and preprocessing models proposed for streaming data applications in real time. In this way, the IDS proposed would be able to detect real attacks with features related to each one of the datasets evaluated during this research. Finally, as a future work, we will continue this research, using a complete version of the UGR16 and big data technologies, taking some considerations from related researches such as the amount of redundant records of the datasets used in this article.

## Figures and Tables

**Table 1:** Proportions of attacks and no attack for UGR16 dataset.

Category	Percentage
Attack	12.82%
No Attack	87.18%

**Table 2:** Proportions of attacks and no attack for UNSW-NB15 dataset.

Category	Percentage
Attack	44.94%
No Attack	55.06%

**Table 3:** Proportions of attacks and no attack for NSL-KDD dataset.

Category	Percentage
Attack	46.54%
No Attack	53.56%

**Table 4:** Confusion matrix for the actual class vs. the predicted class.

	Predicted Class	
Actual Class	TP	FN
	FP	TN

**Table 5:** Categorical data transformations for the datasets understudy.

Dataset	One-Hot Encoding	Binary Vector
UGR16	protocol, flag	attack_tag
NSL-KDD	protol_type, service, flag, land, num_failed_login, is_host_login, is_guest_login	attack_tag
UNSW-NB15	dur, proto, service	attack_tag

**Table 6:** Variations in terms of accuracy according to preprocessing techniques for the NSL-KDD dataset.

Basic Characteristics	Content Characteristics	Flow Characteristics	Accuracy
z-score	min_max_0	z-score	0.97923414
min_max_0	min_max_0	z-score	0.978567346
z-score	min_max_0	min_max_0	0.97694799
min_max_0	z-score	z-score	0.974693592
z-score	z-score	min_max_0	0.971550137
min_max_0	z-score	min_max_0	0.970851591
min_max_0	-	z-score	0.970121293
min_max_0	z-score	z-score	0.969867276
z-score	z-score	min_max_0	0.964628183
min_max_0	z-score	-	0.946434241

**Table 7:** Variations according to preprocessing techniques for the UNSW-NB15 dataset.

Basic Characteristics.	Flow Characteristics	Content Characteristics	Direction-Based Traffic Characteristics	Accuracy
z-score	z-score	z-score	min_max_0	0.992
z-score	min_max_0	min_max_0	min_max_0	0.9908
z-score	-	-	-	0.8638
z-score	z-score	z-score	-	0.8618
		z-score	-	0.8349
-	z-score	z-score	z-score	0.7869
-	z-score	z-score	-	0.7163
-	-	-	z-score	0.6581
-	z-score	-	-	0.5775

**Table 8:** Variations according to preprocessing techniques for the proposed datasets.

Configuration	Dataset	Basic Characteristics	Content Characteristics	Flow Characteristics	Direction-Based Traffic Characteristics	Accuracy
N01	NSL-KDD	z-score	min_max_0	z-score		0.997
N02	NSL-KDD	min_max_0	min_max_0	z-score		0.978
N03	NSL-KDD	z-score	z-score	z-score		0.978
N04	NSL-KDD					0.95
N05	UGR16	z-score				0.993
N06	UGR16					0.87
N07	UNSW-NB15	z-score	z-score	z-score	min_max_0	0.992
N08	UNSW-NB15	min_max_0	min_max_0	z-score	min_max_0	0.990
N09	UNSW-NB15	z-score	z-score	z-score	z-score	0.983
N10	UNSW-NB15					0.55

**Table 9:** Confusion Matrix in terms of percentage for the most accurate models.

		Model N01		Model N05		Model N07	
True Label	Attack	47.94%	0.03%	11.55%	0.49%	43.31%	0.39%
	No Attack	0.36%	51.67%	0.14%	87.82%	1.18%	55.12%
		Attack	No Attack	Attack	No Attack	Attack	No Attack
		Predicted Label					

**Table 10:** Recall and Precision for the most accurate models presented during this research.

	Model N01	Model N05	Model N07
Precision	0.992	0.988	0.973
Recall	0.999	0.959	0.990

**Table 11:** Variations according to preprocessing techniques for the proposed datasets.

Research and Configuration Proposed	Preprocessing Technique			ML Model Applied	Dataset	Accuracy
	Scalar	Normalization	Categorical			
N01-Model proposed	Yes	Yes	Yes	NN	NSL-KDD	0.997
N05-Model proposed	Yes	Yes	Yes	NN	UNSW-NB15	0.992
N07-Model proposed	Yes	Yes	Yes	NN	UGR16	0.993
Paulauskas et al.	No	Yes	No	NN	NSL-KDD	0.99
Salih et al.	No	Yes	No	NN	NSL-KDD	0.989
Lokeswari et al.	Yes	No	Yes	NN	KDD99	0.99
Chiba et al.	Yes	Yes	No	NN	KDD99	0.98
